# 

**Published:** 1882-07

**Authors:** 


SURG GENLS OFFS

Vol. XIX. July, 1882. No. 2.

### THE BISTOURY.

Thad S. Up de Graff, M.D.
EDITOR.

ELMIRA, N.Y.

